# Does Interactive Imagery Influence the Reactive Effect of Judgments of Learning on Memory?

**DOI:** 10.3390/jintelligence11070139

**Published:** 2023-07-13

**Authors:** Amber E. Witherby, Addison L. Babineau, Sarah K. Tauber

**Affiliations:** 1 Department of Psychological Sciences, Creighton University, Omaha, NE 68178, USA; 2 Department of Psychology, Texas Christian University, Fort Worth, TX 76129, USA; a.babineau@tcu.edu (A.L.B.); uma.tauber@tcu.edu (S.K.T.)

**Keywords:** judgments of learning, judgment reactivity, metamemory, mental imagery

## Abstract

Making judgments of learning (JOLs) while studying is a useful tool for students to evaluate the status of their learning. Additionally, in associative learning contexts, JOLs can directly benefit learning when the to-be-learned information is related. One explanation for this reactive effect is that making JOLs strengthens the associative relationship, leading to enhanced memory performance when a test relies on that relationship (e.g., cued-recall tests). In the present research, we evaluated whether having students make interactive mental images—another strategy that can increase the strength of a cue–target relationship—impacts the reactive effect of JOLs on learning. Students studied word pairs that were related and unrelated. Half of the students were instructed to form a mental image of the words interacting, whereas the other half were not. Additionally, in each group half of the students made a JOL for each pair, whereas half did not. Following a short delay, students completed a cued-recall test. Consistent with prior research, students who made JOLs remembered more related word pairs than did students who did not. By contrast, students who made JOLs recalled fewer unrelated word pairs than did students who did not. Moreover, although students who formed interactive images demonstrated enhanced memory relative to students who did not, interactive imagery did not impact the reactive effect of JOLs. These outcomes are informative for existing theory of JOL reactivity. Specifically, JOLs may only benefit learning of associative information when it has a pre-existing semantic relationship (e.g., related word pairs) and not when that that relationship is created by the learner (e.g., by forming interactive images).

## 1. Introduction

When studying for a test, a useful starting place for students is to evaluate the status of their memory for the to-be-tested information. For instance, students can make judgments of learning (JOLs) by predicting the likelihood that they will remember each piece of information on the test. By doing so, students can strategically regulate their learning to focus on the information judged to be the least well known (i.e., the items with low JOLs), which should benefit their later test performance. Indeed, ample evidence supports this indirect effect of students’ monitoring judgments (i.e., memory assessments such as JOLs) on later test performance (e.g., Dunlosky and Hertzog 1997; Tauber and Rhodes 2012; Thiede 1999). More recently, researchers have become interested in the potential direct impact that JOLs might have on learning (i.e., JOL reactivity). That is, can simply making JOLs during learning directly impact later retention of the studied information? The answer to this question, based on existing research, is sometimes. Our primary goal was to evaluate when, and why, JOLs have positive reactive effects on memory.

Although JOLs are often assumed to be innocuous measures of monitoring accuracy (i.e., people’s ability to evaluate the status of their memory), researchers have speculated that they might reactively affect memory (e.g., Spellman and Bjork 1992). This possibility was first experimentally tested by Soderstrom et al. (2015). Participants studied a list of related word pairs (e.g., *loaf–bread*) and unrelated word pairs (e.g., *sack–flag*) each presented for 8 s. Half of the participants made a JOL for each word pair, predicting the likelihood that they would remember the second word of the pair (e.g., *bread*) when given the first (e.g., *loaf–?*) on a later cued-recall test. Participants made their JOL midway through studying each pair and had the remainder of the 8 s to make their judgment. The other half of the participants studied each word pair without making any judgments. After a short 3 min retention interval, participants completed a cued-recall test. For unrelated word pairs, memory performance did not differ between participants who made JOLs and those who did not. By contrast, for related word pairs, participants who made JOLs recalled significantly more than did those who did not. Witherby and Tauber (2017) replicated this positive reactive effect of JOLs on memory for related word pairs and extended it by showing that the memory benefit persists even after a longer 2-day retention interval. Moreover, meta-analyses across experiments revealed that the positive reactive effect of JOLs on memory for related word pairs was large after both a 3 min retention interval (*d* = .71) and a 2-day retention interval (*d* = .66).

Although JOLs appear to benefit memory for related word pairs (e.g., Li et al. 2022; Rivers et al. 2023; Tauber and Witherby 2019), they typically either have no effect, or sometimes even a negative effect, on memory for unrelated word pairs (e.g., Chang and Brainerd 2023; Dougherty et al. 2018; Halamish and Undorf 2023; Maxwell and Huff 2022, 2023; Mitchum et al. 2016; Myers et al. 2020; Janes et al. 2018; Rivers et al. 2021; Senkova and Otani 2021; Soderstrom et al. 2015; see Double et al. 2017 for a meta-analysis). To illustrate, using a design such as that of Soderstrom and colleagues, Mitchum et al. (2016, Exp 5) had participants study a list of related and unrelated word pairs for a cued-recall test. Half of the participants made a JOL for each and half did not. Consistent with prior outcomes, participants who made JOLs recalled more related word pairs compared to participants who did not. By contrast, participants who made JOLs recalled fewer unrelated word pairs compared to participants who did not. A significant interaction revealed that the requirement to make JOLs increased the size of the relatedness effect on memory when compared to participants who did not have to make JOLs (see also Janes et al. 2018).

According to the *cue-strengthening* account proposed by Soderstrom et al. (2015), related word pairs have an inherent relationship (e.g., the words may be semantically related) and participants use this relationship to inform their JOLs. Further, the act of making a JOL strengthens that existing relationship. As a result, when participants complete a test that is sensitive to that relationship (e.g., a cued-recall test), memory will be enhanced if they made JOLs compared to if they did not.^1^ By contrast, unrelated word pairs do not have an inherent relationship, and as such, there is no relationship to be strengthened by the act of making a JOL. Several studies have tested various predictions of this theory, and outcomes have generally supported it. For instance, Myers et al. (2020) tested the prediction that memory will only be enhanced when the test is sensitive to the relationship between the cue and the target. They manipulated the type of memory test participants completed (cued-recall, free-recall, and recognition). Of these tests, cued-recall performance is most likely to be sensitive to cue–target relationships, because participants are given the cue on the test to facilitate recall of the target. By contrast, free recall and item recognition rely less on the cue–target relationships because the cue is not available to participants when retrieving the target. Meta-analyses on the data across all experiments revealed that, consistent with the previously discussed research, JOLs had minimal impact on memory for unrelated word pairs regardless of type of test. However, for related word pairs, test format significantly moderated the reactive effect of JOLs on memory. Specifically, a strong positive reactive effect of JOLs was evident for cued-recall tests (*d* = .52), a weak positive reactive effect of JOLs on memory was evident for item recognition tests (*d* = .23), and no JOL reactivity was evident for free-recall tests (*d* = −.04). Thus, consistent with cue-strengthening theory, positive JOL reactivity was strongest when the test was sensitive to cue–target relationships (i.e., cued-recall).

Researchers have further evaluated the cue-strengthening theory by exploring whether different types of relationships—as compared to strongly related word pairs—result in positive JOL reactivity (i.e., higher memory performance when JOLs are made compared to when they are not). The results of these studies have been largely consistent with the predictions of cue-strengthening theory. For instance, researchers have found positive JOL reactivity for weakly related word pairs (e.g., *dairy–cow*; Tauber and Witherby 2019), backwards related word pairs (e.g., *card–credit*; Maxwell and Huff 2022), symmetrically related word pairs (i.e., forward and backward strength are identical, such as *ball–bounce*; Maxwell and Huff 2022), and identical word pairs (e.g., kiss–kiss; Halamish and Undorf 2023). As another example, Rivers et al. (2023) evaluated whether JOLs result in reactive effects on memory for a two novel types of relationships: letter-cued pairs (e.g., *ja–jade*) and category-cued pairs (*a type of gem–Jade*). In this context, category-cued pairs share a semantic relationship, whereas letter-cued pairs do not, but are related such that the cue is the first two letters of the target word. As in prior experiments, participants studied each item and either made a JOL or they did not. Participants then completed a cued-recall test on which they were presented with the same cues from the study phase (i.e., letter cue or category cue) and had to retrieve the target word that was paired with it. JOLs resulted in positive reactivity for category-cued pairs but did not impact memory for letter-cued pairs (using a different design, Kubik et al. (2022) found positive reactivity for letter-cued pairs). The outcomes of this study provide evidence that the type of relationship can play an important role in whether reactivity will be observed.

A final assumption of cue-strengthening theory is that making JOLs will strengthen the relationship between a cue and a target, presumably by affecting how that relationship is processed. Halamish and Undorf (2023) evaluated this *relatedness processing assumption*. To do so, participants completed the same general paradigm as outlined previously: participants studied a list of related, unrelated, and identical word pairs, half of the participants made a JOL for each and half did not, and then they took a cued-recall test. Critically, on the test, participants made a relatedness recognition decision for each item. Specifically, before retrieving the target for each pair, participants had to decide whether the cue was originally presented with a related target, an unrelated target, or an identical target. After making the recognition decision, participants attempted to retrieve the target. Their outcomes supported the relatedness processing assumption. Specifically, participants who made JOLs performed better on the relatedness recognition task compared to participants who did not. Moreover, this effect held even when limiting the analysis to unrecalled unrelated word pairs, which rules out the possibility that this outcome was a byproduct of memory performance (i.e., if you can remember the target, you can use it to make the recognition decision). Rather, it supports the prediction that participants who make JOLs process the relationship differently compared to participants who do not, leading to more accurate memory for the existing relationship (even when they are unable to retrieve the exact target).

In sum, previous research has largely supported the main tenets of cue-strengthening theory. Even so, these studies have exclusively focused on items that either had an existing a priori relationship or did not. Thus, an open question is whether JOLs will also have reactive effects on memory when participants create relationships between items rather than relying on pre-existing relationships. The goal of the present research was to answer this question. From cue-strengthening theory, if participants create a relationship between two words and they rely on that relationship when making JOLs, then memory should be enhanced when they complete a test that is sensitive to that relationship. In the present experiment, we tested this prediction. Specifically, participants studied a list of related and unrelated word pairs. Half of the participants made a JOL for each word pair and half did not. Critically, half of the participants were instructed to create a relationship between the cue and target words by forming a mental image of the two words interacting (cf. Dunlosky and Hertzog 1997; Hertzog et al. 2003, 2013). The other half of the participants were not given any strategies to use during encoding.

Based on the existing literature, our hypotheses for the no-imagery groups were straightforward. Specifically, we expected to observe positive JOL reactivity for related word pairs and no reactivity (or slight negative reactivity) for unrelated word pairs. For the imagery groups, based on cue-strengthening theory, we expected to see positive JOL reactivity for both related and unrelated word pairs. Moreover, we expected this to be especially likely if participants used imagery as a basis for their JOLs, which would be evident if the magnitude of JOLs was higher for participants in the imagery groups compared to participants in the no-imagery groups.

## 2. Materials and Methods

### 2.1. Design and Participants

A 2 (judgment: no JOL, JOL; between-participants) × 2 (imagery: no imagery, imagery; between-participants) × 2 (word type: related, unrelated; within-participants) mixed design was implemented. Using the effect size of *d* = .71, (Witherby and Tauber 2017; mini meta-analysis estimating a reactive effect of JOLs on immediate test performance) with alpha at .05, and power at .95, a power analysis using G*Power estimated that 43 participants were necessary for each group (Faul et al. 2007). Accordingly, 176 participants completed the study and were randomly assigned to one of the between-participants groups: JOL with imagery, *n* = 45; JOL without imagery, *n* = 44; no-JOL with imagery, *n* = 44; no-JOL without imagery, *n* = 43.

Participants were college-aged (JOL with imagery, *M* = 19.69 years, *SE* = .32; JOL without imagery, *M* = 19.52 years, *SE* = .31; no-JOL with imagery, *M* = 19.00 years, *SE* = .21; no-JOL without imagery, *M* = 19.09 years, *SE* = .18) and most participants identified as women (JOL with imagery, *n* = 38, 84.4%, 7 men; JOL without imagery, *n* = 27, 61.4%, 17 men; no-JOL with imagery, *n* = 26, 59.1%, 18 men; no-JOL without imagery, *n* = 33, 76.7%, 10 men) and white (JOL with imagery, *n* = 33, 73.3%, 11 Hispanic and/or Latino and 1 Asian and/or Pacific Islander; JOL without imagery, *n* = 32, 72.7%, 4 Hispanic and/or Latino, 4 Asian and/or Pacific Islander, 3 black, and 1 black and Asian; no-JOL with imagery, *n* = 26, 59.1%, 9 Hispanic and/or Latino, 4 black, 3 Asian and/or Pacific Islander, and 2 Native American; no-JOL without imagery, *n* = 30, 69.8%, 6 Asian and/or Pacific Islander, 5 Hispanic and/or Latino, 2 black). The groups did not significantly differ on age, *F*(3, 172) = 1.61 and *p* = .19, or ethnicity, *χ*^2^ (3) = 21.95 and *p* = .11.^2^ There was a significant difference in gender identity between groups, *χ*^2^ (3) = 9.53, *p* = .023; although most participants in each group identified as women, more participants in the JOL with imagery group identified as women than did those in the JOL without imagery group, *χ*^2^ (1) = 5.03, *p* = .025, and the no-JOL with imagery group, *χ*^2^ (1) = 7.08, *p* = .008.

### 2.2. Materials and Procedure

Participants studied 30 related word pairs and 30 unrelated word pairs. The 30-item list of related word pairs (modified from Tauber and Witherby 2019) consisted of related concrete nouns (e.g., couch—potato) from the Nelson et al. (2004) free-association norms (associated relatedness *M* = .15, *SD* = .03).^3^ The related cue and target words did not differ in length, *t*(29) = 1.70 and *p* = .10, frequency, *t*(29) = 1.84 and *p* = .08, or the number of syllables, *t*(29) = 1.77 and *p* = .86. The 30-item list of unrelated word pairs (e.g., arrow—island) was generated such that the unrelated cue and target words were both concrete nouns, were not listed as semantic associates of each other in Nelson et al.’s (2004) norms, and did not differ in length, *t*(29) = 1.65, *p* = .11, frequency, *t*(29) = .55, *p* = .59, or the number of syllables, *t*(29) = .72, *p* = .48. Further, the length, *F*(3, 87) = 1.57, *p* = .20, frequency, *F*(3, 87) = 2.15, *p* = .10, and the number of syllables, *F*(3, 87) = .25, *p* = .86, of the words did not significantly differ between the related word pairs and unrelated word pairs.

Prior to study, participants in the imagery groups were given detailed instructions to use interactive imagery when encoding each word pair. Specifically, we adopted a procedure that has been validated in past work, such that participants were successful at forming interactive images for most items and the requirement to form interactive images was related to participants’ monitoring judgments (Dunlosky and Hertzog 1997; Hertzog et al. 2003, 2013). For each word pair, participants in the imagery group were asked to form a mental image of the cue word and target word interacting. Additionally, participants were given two word pairs that did not appear in the study list to provide examples of how to develop interactive mental images (“For example, if you saw the pair Crown-King, you could image a crown on a king’s head. It is also ok if your picture is uncommon or seems bizarre. For example, if you saw the pair Cowboy-Duck, you could imagine a cowboy riding on a duck. The most important thing is that for each pair of words that you see, you should make one mental image of the two concepts interacting.”). Participants in the imagery groups also completed two practice trials to familiarize themselves with forming mental images of the words interacting. Participants in the no-imagery groups were not instructed to use any specific strategy during learning.

Prior to study, participants in the JOL groups were instructed to study each word pair for 4 seconds, after which they would make a judgment of how likely they are to remember it on a memory test in about 10 minutes. The JOL groups completed two practice trials to familiarize themselves with this JOL procedure.

Participants in all groups studied all 60 word pairs one-at-a-time, and each word pair was presented for 8 s. The order of the word pairs was random for each participant. After studying each word pair for 4 s, participants in the JOL groups were presented with a field beneath each word pair and were instructed to enter their JOL. Participants were given the remaining 4 s of the presentation time to enter their judgment. JOLs were made on a scale of 0% to 100%. A JOL of 0% indicated that the participant was certain they would not recall the word pair on the test and a JOL of 100% indicated the participant was certain they would recall the word pair on the test. Participants in the no-JOL groups studied the word pair for the entire 8 s with no JOL prompts. Following presentation of all 60 word pairs, all groups completed a brief 3 min distractor task. Next, participants in all groups completed the cued-recall test. During the test, participants were presented with the first word from each word pair and were asked to enter the second word in the pair. Each item was presented one-at-a-time and the test was self-paced. The order of pairs on the test was randomized for each participant and feedback was not provided.

## 3. Results

### 3.1. Cued-Recall Performance

**Figure 1:**
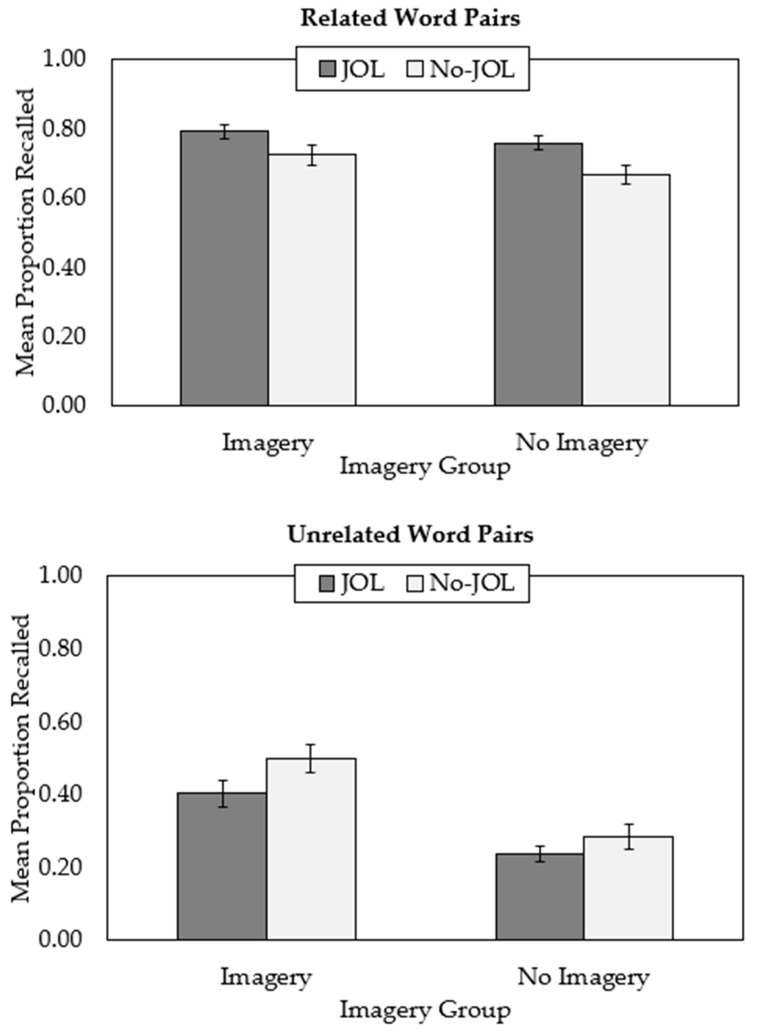
Mean proportion correctly recalled for related word pairs (**top** panel) and unrelated word pairs (**bottom** panel) in the present experiment. JOL = judgment of learning. Error bars represent one standard error of the mean.

The outcomes of the present experiment replicated previous work showing positive JOL reactivity for related word pairs (Figure 1, top panel) and negative reactivity for unrelated word pairs (Figure 1, bottom panel), though this latter effect was smaller and only marginally significant). Although recall was superior for participants who were instructed to use imagery compared to participants who were not, this manipulation did not interact with JOL condition to impact the reactive effects of JOLs.

These outcomes were confirmed with a 2 (judgment: no-JOL, JOL) × 2 (imagery: no imagery, imagery) × 2 (word type: related, unrelated) mixed analysis of variance (ANOVA). A main effect of word type, indicating that participants recalled more related word pairs (*M* = .74, *SE* = .01) relative to unrelated word pairs (*M* = .36, *SE* = .02), *F*(1,172) = 1018.71, *p* < .001, η_p_^2^ = .86, and a non-significant effect of judgment group, *F* < 1, were qualified by a significant two-way interaction between these variables, *F*(1,172) = 39.47, *p* < .001, η_p_^2^ = .19. As evident in Figure 1 (top panel), participants who made JOLs recalled significantly more related word pairs (*M* = .77, *SE* = .01) compared to participants who did not (*M* = .70, *SE* = .02), *t*(174) = 3.21, *p* = .002, *d* = .47. By contrast, for unrelated word pairs, participants who made JOLs (*M* = .32, *SE* = .02) showed marginally worse performance compared to participants who did not make JOLs (*M* = .39, *SE* = .03), *t*(174) = 2.00, *p* = .05, *d* = .30 (see Figure 1, bottom panel). Thus, JOLs had a positive reactive effect on memory for related word pairs, and a small negative reactive effect on memory for unrelated word pairs.

The analysis also revealed a significant main effect of the imagery group, *F*(1,172) = 19.71, *p* < .001, and η_p_^2^ = .10. Specifically, participants in the imagery groups recalled more word pairs (*M* = .60, *SE* = .02) compared to participants in the no-imagery groups (*M* = .49, *SE* = .02). The main effect is expected and establishes the effectiveness of the interactive imagery manipulation for enhancing learning. The two-way interaction between word type and imagery group was also significant, *F*(1,172) = 37.56, *p* < .001, η_p_^2^ = .18. This interaction was driven by the finding that imagery benefited memory for unrelated word pairs but did not impact memory for related word pairs. Specifically, for unrelated word pairs, recall was greater for participants in the imagery groups (*M* = .45, *SE* = .03) relative to participants in the no-imagery groups (*M* = .26, *SE* = .02), *t*(174) = 5.68, *p* < .001, *d* = .79. By contrast, for related word pairs, recall did not differ for participants in the imagery groups (*M* = .76, *SE* = .02) compared to participants in the no-imagery groups (*M* = .71, *SE* = .02), *t*(174) = 1.79, *p* = .08, *d* = .27. The two-way interaction between imagery group and judgment group was not significant, *F* < 1, nor was the three-way interaction, *F* < 1. Thus, the requirement to use imagery as a strategy during learning did not impact the reactive effect of JOLs on learning of related or unrelated word pairs.

### 3.2. Judgment of Learning Magnitude

Consistent with prior research, JOLs were higher for related compared to unrelated word pairs and for participants in the imagery group compared to the no-imagery group (see Table 1). These outcomes were supported with a 2 (imagery: No imagery, imagery) × 2 (word type: Related, unrelated) mixed ANOVA. A main effect of word type, *F*(1,87) = 521.70, *p* < .001, η_p_^2^ = .67, confirmed that JOLs were higher for related (*M* = 79.37, *SE* = 1.42) compared to unrelated (*M* = 41.45, *SE* = 2.26) word pairs. In addition, a main effect of imagery group, *F*(1,87) = 4.20, *p* = .04, η_p_^2^ = .05, indicated that JOLs were higher in the imagery group (*M* = 63.88, *SE* = 2.38) compared to the no-imagery group (*M* = 56.94, *SE* = 2.41). The interaction was not significant, *F*(1,87) = 2.11, *p* = .15, η_p_^2^ = .02.

## 4. Discussion

An abundance of research has demonstrated that JOLs have positive reactive effects on memory for various kinds of related information, whereas they tend to either have negative or no reactive effects on memory for unrelated information (e.g., Chang and Brainerd 2023; Halamish and Undorf 2023; Maxwell and Huff 2022, 2023; Mitchum et al. 2016; Myers et al. 2020; Janes et al. 2018; Rivers et al. 2021; Senkova and Otani 2021; Soderstrom et al. 2015). The new question guiding the present research was, would JOLs have reactive effects on memory when participants created relationships for items that were previously unrelated? Based on the cue-strengthening theory, we anticipated that, by having participants form interactive images for unrelated word pairs, a relationship would be established that could in turn be strengthened by making a JOL (Soderstrom et al. 2015). Moreover, given that the cued-recall test would be sensitive to this relationship, we anticipated positive JOL reactivity for unrelated word pairs for participants in the interactive imagery group. However, the outcomes of the present experiment were inconsistent with this prediction. In fact, we observed negative reactivity for unrelated word pairs (i.e., worse memory performance for participants who made JOLs compared to those who did not) regardless of whether participants were in the interactive imagery group. This outcome is consistent with some existing research (e.g., Halamish and Undorf 2023; Mitchum et al. 2016; Janes et al. 2018). Even so, it is worth noting that this effect was relatively small (*d* = .30) and only marginally significant (*p* = .05). As such, additional research is needed before drawing strong conclusions about negative JOL reactivity in this context. By contrast, positive JOL reactivity was observed for related word pairs in both the imagery and no-imagery groups, and this effect was medium in size (*d* = .47). Imagery also had a positive overall effect on memory (small to medium effect size, *d* = .27), such that participants in the imagery group remembered more related and unrelated word pairs compared to participants in the no-imagery group.

Although it is challenging to interpret null effects, we consider a couple of possible explanations for why interactive imagery failed to result in positive reactivity for unrelated word pairs. One possibility is that the interactive imagery manipulation was unsuccessful—that is, perhaps participants did not create a relationship between the cue and target for unrelated word pairs. There are several pieces of evidence that suggest that this possibility is unlikely. First, there was an overall main effect of imagery on recall. Consistent with prior research, forming interactive images enhanced memory compared to not forming interactive images (e.g., Bower 1970; Bower and Winzenz 1970; Pressley et al. 1988; for a review, see Richardson 1998). Additionally, there was also a main effect of imagery on JOLs, such that participants in the imagery group gave higher JOLs compared to participants in the no-imagery group (cf. Dunlosky and Nelson 1994; but see Rabinowitz et al. 1982; Shaughnessy 1981). Thus, the imagery manipulation directly impacted both memory and JOLs as expected for successful interactive imagery implementation. In addition, prior research using similar materials (i.e., unrelated concrete word pairs) and imagery instructions demonstrated that participants successfully made interactive images for most pairs (e.g., 75–89% of trials, Hertzog et al. 2003) and were able to do so in the amount of time we allotted in the present experiment (e.g., participants in Hertzog et al. (2003) took on average 4.3 s to generate an interactive mental image).

Another possibility relates to a theoretical explanation for negative JOL reactivity: the dual-task account (Mitchum et al. 2016). From this perspective, having participants complete two tasks simultaneously can impair performance if both tasks are resource demanding. This perspective has been used to explain why JOLs fail to benefit memory (and sometimes impair memory) for unrelated word pairs. Specifically, the act of monitoring learning (by making a JOL) might compete for resources with the primary task of learning the word pair. This can be especially problematic when learning items that are difficult (e.g., unrelated word pairs). This dual-task situation is less problematic for easier to learn items (e.g., related word pairs) because learning them is less resource demanding. Thus, this perspective can account for the moderating effect of stimulus type in the reactive effect of JOLs on memory. In the present experiment, having participants create mental images added a third task for them to complete in a relatively short period of time (8 s). Additionally, creating interactive images for unrelated word pairs was likely more resource demanding compared to related word pairs. As such, any additional beneficial effects JOLs might have had on memory for unrelated word pairs may have been attenuated due to the limited mental resources. Future research is needed to test this possibility. For instance, dual-task costs could be attenuated by having participants complete an initial study phase during which they create interactive mental images, followed by a second study phase during which they make JOLs for each item.

There are three primary assumptions from cue-strengthening theory (Soderstrom et al. 2015): (a) participants will use relatedness to inform their JOLs, (b) making JOLs will strengthen that relationship, and (c) JOLs will benefit memory if the test is sensitive to that relationship. The design of the present study presumably met these assumptions. Participants in the imagery groups likely created a relationship for most unrelated word pairs (as evidenced by enhanced memory in the imagery relative to no-imagery group) and participants’ JOLs were influenced by this relationship (as evidenced by higher JOLs in the imagery relative to no-imagery groups). Moreover, we used a test that would be sensitive to these relationships (i.e., cued-recall). Given that these assumptions were met for unrelated word pairs and positive JOL reactivity was not observed, this suggests that the assumptions of this theory may need to be modified. Specifically, the outcomes of the present research suggest that not all relationships will be strengthened by the act of making a JOL. Rather, positive JOL reactivity in associative learning contexts may require existing semantic relationships between the to-be-learned words. Memory for other types of relationships, such as associative relationships formed through interactive imagery (the present experiment), lexical relationships (e.g., letter-cued pairs; Rivers et al. 2023), or other non-semantic relationships, may not benefit from making JOLs via cue-strengthening.

Although the focus of the present research was on testing a specific account of JOL reactivity, other theoretical perspectives have been proposed to explain reactivity effects (or lack of effects). For instance, the *attentional reorienting* account (Tauber and Witherby 2019) suggests that making a JOL (relative to not making one) reorients participants to the word pair, increasing their attention to it, and in turn their memory for it. Specifically, in a no-JOL condition, participants might attend to an item when it is immediately presented, but then over time attention may wane. By contrast, in a JOL condition, participants are reoriented to each item when they are prompted to make a JOL increasing their attention to and processing of it. A similar perspective, the *enhanced learning engagement* account (Shi et al. 2022; Zhao et al. 2022) suggests that JOLs increase engagement with each to-be-remembered item, leading to enhanced memory performance. The *item-order relational* account (Zhao et al. 2023) has been used to explain the moderating effects of item type (e.g., word pairs vs. single words) and test format (e.g., cued-recall vs. free recall) on the reactive effects of JOLs. Specifically, this perspective suggests that JOLs enhance item memory (i.e., memory for target words) and impair memory for relationships between items (e.g., temporal order in which items are presented). In their experiments, they established positive JOL reactivity for recognition tests (which evaluate item memory; see also, Yang et al. 2015), negative reactivity for tests on inter-item relational information (i.e., temporal clustering and order reconstruction), and no-reactivity for free recall tests (which rely on both item memory and interitem memory). As a final example, the *changed-goal* account (Mitchum et al. 2016), suggests that requiring participants to make JOLs highlights the fact that some items will not be remembered. As a result, when making JOLs, participants may change their goals to prioritize learning easier items (e.g., related word pairs) at the cost of more difficult items (e.g., unrelated pairs). This in turn could lead to positive reactivity for easier items and negative reactivity for more difficult items.

Although several theoretical accounts of JOL reactivity have been put forward, it is worth noting they are not mutually exclusive. That is, JOLs may reactively impact memory through a variety of mechanisms, and the extent to which each mechanism contributes will likely depend on the learning conditions. An important goal for future research will be to continue evaluating when and why JOLs impact memory in various contexts. As a path forward in this field, Myers et al. (2020) recommended using the tetrahedral model of memory (Jenkins 1979) for understanding the various moderating factors that impact JOL reactivity. From this model, memory can be influenced by a combination of four factors: item characteristics (e.g., related versus unrelated word pairs), participant characteristics (e.g., prior knowledge), encoding characteristics (e.g., instructions during learning), and retrieval characteristics (e.g., the type of test). Several studies have been conducted evaluating various aspects of this model. For instance, regarding participant characteristics, the bulk of the work on JOL reactivity has focused on younger adults. In the few studies conducted with other age groups, Tauber and Witherby (2019) found no reactivity in older adults on a cued-recall test for related word pairs, and Zhao et al. (2022) found positive reactivity in children on an item recognition test. Regarding encoding conditions, Tekin and Roediger (2020) used the levels of processing approach to evaluate JOL reactivity. Specifically, encoding conditions were manipulated such that participants made a perceptual judgment, a rhyming judgment, or a semantic judgment. Half of the participants made JOLs, and half did not. Most important, the requirement to make JOLs attenuated the levels of processing effect, such that JOLs had stronger reactive effects following shallow processing tasks (i.e., perceptual judgments) compared to deeper processing tasks (i.e., semantic judgments). Similarly, other researchers have shown that the reactivity effects extend to other judgments such as judgments of forgetting (i.e., *how likely are you to forget this item?*; Murphy et al. 2023) and judgments of relatedness (Maxwell and Huff 2022). In a study looking at both the encoding and retrieval conditions, Chang and Brainerd (2023) found that list-level JOLs (i.e., how many items do you think you will remember from the list you just studied) enhanced free-recall, but not cued-recall, whereas item-level JOLs (as in the present experiment) enhanced cued-recall, but not free recall. Finally, other research has focused on the reactive effects of JOLs when learning material other than single words and word pairs. For instance, Shi et al. (2022) found positive JOL reactivity when participants learned pictures of objects and scenes, Ariel et al. (2021) found no JOL reactivity when participants learned text passages (see also, Ha and Lee 2023), and Schafer and Undorf (2023) found no JOL reactivity when participants learned general knowledge facts. In addition, Lee and Ha (2019) found that category-level JOLs resulted in positive reactivity when learning paintings, whereas item-level JOLs did not affect learning. As can be seen by this growing literature, the relationship between JOLs and memory is complex, and many factors can impact whether JOLs will have reactive effects on memory. We encourage additional research in this area, perhaps guided by the tetrahedral model of memory to understand how the reactive effect of JOLs are influenced by various components of this model.

In addition to the theoretical implications discussed previously, another reason researchers have been interested in JOL reactivity is because of the potential applied implications (e.g., Ariel et al. 2021; Schafer and Undorf 2023). Specifically, given that JOLs have been shown to improve memory in some contexts in laboratory studies, it is possible that they could also be used in educational settings to improve learning. Although the goal of the present research was not to test this possibility, the outcomes do suggest that JOLs are unlikely to benefit learning unless there is a pre-existing semantic relationship between the to-be-learned items. That is, it is unlikely that JOLs will have additional positive effects on memory if students create their own relationships for the to-be-learned information (e.g., via interactive imagery).

Finally, as with all research, there are some limitations of this study that can be addressed with future research. One of our key manipulations was the requirement for participants to generate interactive images. Although the data demonstrate that participants in the imagery groups followed these instructions (i.e., significant main effect of imagery on memory performance and JOLs), we did not collect information about the images they generated. It is possible that the quality of participants’ images differed from person to person, as well as from item to item. Moreover, the quality of their images may impact whether interactive imagery moderates the reactive effect of JOLs on memory for unrelated word pairs. Thus, future research conceptually replicating the present research while also collecting data on participants’ image generation will be useful for better understanding the relationship between interactive imagery and the reactive effects of JOLs. In addition, the novel finding of this study (i.e., that interactive imagery does not impact the reactive effect of JOLs on learning) is based on a null effect (i.e., no interaction between JOL group and imagery group). A null result can be challenging to interpret because it is possible that the effect truly does not exist, or the study was not sensitive enough to detect it. Although we conducted an a priori power analysis to help circumvent the latter possibility, it is still possible that the outcomes could be due to a type II error. Thus, future research replicating this experiment with a larger sample size will be useful for attenuating this concern.

## 5. Conclusions

In sum, the present research demonstrated a boundary effect for the reactive effect of JOLs on memory for related information. Specifically, the outcomes suggest that JOLs will only benefit learning of related information when the to-be-learned items have a pre-existing relationship or when the relationship is semantic in nature. By contrast, associative relationships created by forming interactive mental images do not appear to be strengthened by making JOLs.

## Figures and Tables

**Table 1:** Judgment of learning magnitude in the present experiment.

Group	Related Word Pairs	Unrelated Word Pairs
Imagery	81.63 (2.01)	46.13 (3.69)
No Imagery	77.11 (2.05)	36.78 (3.07)

Note. Values represent mean judgments of learning (JOLs) for each group. Standard errors of the mean are in parentheses.

## Data Availability

All materials and raw data have been uploaded to the Open Science Framework and can be accessed at https://osf.io/q87g2/?view_only=93d422e3c367445a9e211c562e8be87b.

## Notes

1 Cue-strengthening theory is the predominant theory that has been used to explain JOL reactivity in associative learning contexts, and it is the theory we used to guide the current research. Other theories have been proposed to explain JOL reactivity in other contexts, and we discuss those in the General Discussion.

2 We used the Statistical Package for Social Sciences (SPSS) for these and all remaining statistical analyses.

3 The exact materials used in the present experiment can be found on the Open Science Framework, located at https://osf.io/q87g2/?view_only=93d422e3c367445a9e211c562e8be87b.
